# The Role of Gut Microbiota in Neurodegenerative Diseases: Current Insights and Therapeutic Implications

**DOI:** 10.7759/cureus.47861

**Published:** 2023-10-28

**Authors:** Arpit Jain, Suryansh Madkan, Praful Patil

**Affiliations:** 1 Anatomy, Jawaharlal Nehru Medical College, Datta Meghe Institute of Higher Education and Research, Wardha, IND; 2 Ophthalmology, Maharishi Markandeshwar University, Ambala, IND; 3 Microbiology, Jawaharlal Nehru Medical College, Datta Meghe Institute of Higher Education and Research, Wardha, IND

**Keywords:** amytrophic lateral sclerosis, dysbiosis, gut microbiota, alzheimer's disease, parkinson' s disease

## Abstract

Small microscopic entities known as microbes, having a population of hundreds of billions or perhaps even in trillions, reside in our gastrointestinal tract. A healthy immune system, digestion, and creation of vitamins and enzymes are all thanks to these microbes. However, new research has shown a hitherto unrecognized connection between the microbiota of the intestines and the genesis of neurodegenerative diseases. Neurons in the CNS gradually deteriorate in neurodegenerative illnesses like multiple sclerosis and Parkinson's disease (PD). This deterioration impairs cognitive and physical function. Amyotrophic lateral sclerosis (ALS), PD, and Alzheimer's disease (AD) are just a few examples of neurodegenerative illnesses that pose a serious threat to world health and have few effective treatments. Recent research suggests that the gut microbiota, a diverse microbial population found in the gastrointestinal system, may substantially impact the cause and development of various diseases. The discovery of altered gut microbiota composition in people with these illnesses is one of the most critical lines of evidence connecting gut microbiota dysbiosis to neurodegenerative diseases. AD patients have a distinct characteristic of having a particular microbiota profile. In addition, an excess population of a specific microbe data profile is seen as compared to a healthy individual. Similar changes in the gut microbiota composition have been noted in people with multiple sclerosis and PD. The latest study indicates the potential that dysbiosis, a condition characterized by alteration in the intestinal microbiota's makeup and functioning, may have an effect on the onset and progression of neurodegenerative diseases, including PD and multiple sclerosis. In order to emphasize any potential underlying mechanisms and examine potential treatment repercussions, the review article's goal is to summarize current knowledge about the connection between gut microbiota and neurodegenerative disorders. The review article aims to summarize current knowledge about the connection between gut microbiota and neurodegenerative disorders, highlighting potential underlying mechanisms and examining potential treatment repercussions.

## Introduction and background

The gut microbiome is a vast community composed of billions of microorganisms that play a significant role in the smooth operation of human life. Research has shown the influence of gut microbiome on disease influenced by gut microbes. Furthermore, experimental studies in animal models have further supported intestinal microbes' function in neurological conditions. For instance, studies indicate that compared to rodents bred traditionally, pathogen-free mice, which lack intestinal microbes, display reduced neuroinflammation and enhanced cognitive function. These data imply that dysbiotic gut microbiota may factor in several diseases like neuroinflammation and neuronal dysfunction observed in neurodegenerative diseases. The "gut-brain axis" theory emphasizes the network that links the gastrointestinal tract and the brain in both directions. This complex interaction comprises hormonal, immunological, immunological, and microbial pathways that collectively affect biological processes while affecting the health of the intestinal tract and the brain [1].

Through the microbiota-gut-brain axis, gut dysbiosis, a significant root cause in numerous gastrointestinal illnesses, may also increase pro-inflammatory cytokines, monocytes, lipopolysaccharides, and T helper cells, promoting intestine and the permeability of the blood-brain barrier (BBB). As a result, the progression of neurodegenerative illnesses, including amyotrophic lateral sclerosis (ALS), Alzheimer's disease (AD), and Parkinson's disease (PD), is accelerated. These diseases encompass demyelination, axonal damage, and multiple sclerosis. According to studies, consuming probiotics may aid in the integrity of the intestine and BBB, ameliorating the aforementioned neurodegenerative illnesses [1]. Through the microbiota-gut-brain axis, microbes mediate communication between the metabolic, peripheral immunological, and central neurological systems. However, it is uncertain how the association between the microbiota of the gut and brain cells works or how this relationship affects intellectual and normal brain function [2]. The gut bacteria is being linked to more clinical and preclinical research as a potentially significant risk factor for diseases of the nervous system like autism, multiple sclerosis, stroke spectrum disorder, AD, and PD [3]. Inflammation, oxidative stress, and loss of gut barrier integrity have all been linked to the etiology of neurodegenerative diseases. New research reveals that changes in the makeup of the gut microbiota may play a role in these conditions.

The control of immune responses, the production of metabolites that may impact brain health, and the accumulation of proteins linked to the disease have also been associated with specific bacterial species. For example, compared to normal subjects, those with PD and AD have less diversity of microbes and different microbial makeup. Furthermore, it has been discovered that people with neurodegenerative diseases have drastically changed levels of particular bacteria, including Firmicutes and Bacteroidetes. With the inclusion of the complex world of gut microbiota, these discoveries have prompted a rethinking of the factors that contribute to neurodegenerative illnesses, going beyond just hereditary and environmental factors. Modern developments in metagenomic analysis and high-throughput sequencing technology have sparked research into the relationship between intestinal microbes and neurological disorders. These approaches have given researchers previously unheard-of insights into the richness and makeup of the gut microbiota, enabling them to pinpoint specific microbial taxa and functional pathways linked to neurodegenerative diseases. For instance, research has shown that people with PD and AD have altered gut microbiotas, showing variations in the abundance of particular bacterial taxa and the possibility of metabolic pathway dysregulation [4,5]. It is possible that neurodegenerative diseases affect intestinal-innervating nerve cells and CNS neurons because they disrupt vital CNS processes as well as persistent gut dysfunctions. Despite the CNS physiology of neurodegenerative diseases still being extensively researched, little is known about how intestine-innervating nerve cells, such as those that link the brain to the intestines, are impacted by or involved in the etiology of these disabling and degenerative illnesses [6].

To explain how gut microbiota can affect neurodegenerative illnesses, several hypotheses have been put forth. First off, dysbiosis can result in widespread inflammation, resulting in neuroinflammation. Gut bacteria play a critical role in regulating immune system activity. Second, different metabolites produced by gut bacteria, including short-chain fatty acids (SCFAs) and neurotransmitters, can have an impact on the health and function of the brain. The gut microbiota may additionally influence the BBB's integrity, which can lead dangerous neurotoxins to enter the CNS. These results highlight not only the intestinal microbiota's potential relevance but also the significance of unique microbial profiles that may influence illness vulnerability and development. The therapeutic possibilities of using the microbiota of the gastrointestinal tract to control or perhaps prevent neurodegenerative illnesses are gaining traction in light of these fascinating results. A new route for intervention has opened up with the idea of "psychobiotics," living microorganisms that may have psychological advantages. In order to reduce inflammation, strengthen the gut barrier, and possibly slow the advancement of neurodegenerative diseases, probiotics, prebiotics, and dietary interventions that modify the composition of the intestinal microbiota and encourage the growth of beneficial microbes have been investigated [7,8].

## Review

Methodology

**Figure 1:**
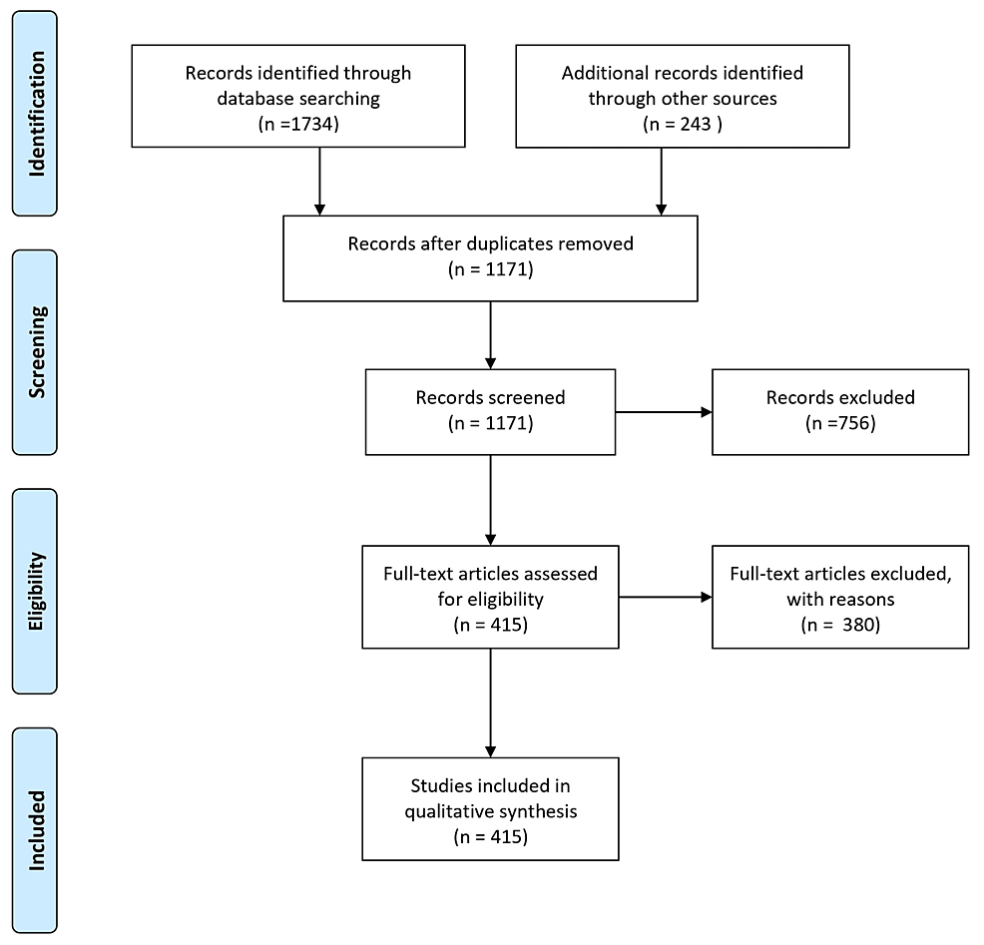
The selection process of articles used in the study Adopted from the Preferred Reporting Items for Systemic Reviews and Meta-Analyses (PRISMA)

A comprehensive literature search strategy was implemented to identify relevant studies investigating the role of gut microbiota in neurodegenerative diseases. Electronic databases, including PubMed/MEDLINE, Embase, and Google Scholar, were searched using keywords and Medical Subject Headings (MeSH) terms. The search was limited to articles published within the last 10 years without language restrictions. The reference lists of relevant articles and review papers were manually screened to identify additional studies. To ensure the inclusion of appropriate studies, specific inclusion criteria were applied. Eligible studies are needed to focus on the role of gut microbiota in neurodegenerative diseases. Studies unrelated to the research question, those focusing on non-human subjects or in vitro/experimental models, and articles lacking sufficient data or available only as conference abstracts, editorials, commentaries, or opinion articles were excluded. The selection process involved screening the titles and abstracts of identified articles, followed by a full-text assessment based on the inclusion and exclusion criteria. Any disagreements or uncertainties were resolved through discussions among the authors. By implementing these rigorous methodology steps, we aimed to ensure the inclusion of high-quality and relevant studies in this review article, thus providing a comprehensive and reliable overview of the role of gut microbiota in neurodegenerative diseases. Figure 1 describes the selection process of articles used in our study.

Introduction to gut microbiota and neurodegenerative diseases

The human body, a highly orchestrated symphony of biological processes, is home to a diverse variety of microorganisms, the majority of which reside in the digestive system. The gut microbiota is an amalgam of bacteria, viruses, fungi, and other microscopic life forms that coexist peacefully with the host organism, humans, affecting a wide range of biological processes besides conventional digestion and metabolism [9]. The gradual destruction of neurons and brain connections, on the other hand, is a hallmark of neurodegenerative illnesses like PD and AD. Cognitive decline and memory loss are symptoms of AD, characterized by the buildup of beta-amyloid plaques and tau tangles in the brain. On the other hand, PD results in tremors and motor dysfunction because dopamine-producing neurons degenerate and create alpha-synuclein aggregates [10]. Recent studies have shed light on the possible role of the microbiota of the gut in the emergence of these disorders, even if the main processes underlying neurodegeneration are still being studied.

Neurodegenerative diseases: an overview

A category of chronic, progressive conditions known as neurodegenerative diseases is characterized by the slow degeneration of neurons in the CNS. These disorders frequently result in crippling cognitive, physical, and psychological problems and ultimately result in the loss of certain brain functions. PD, ALS, and AD are three of the most common neurodegenerative illnesses. Each has unique clinical characteristics, pathological signatures, and underlying mechanisms.

Parkinson's Disease

The loss of dopamine-producing neurons in the substantia nigra region of the brain causes PD, a disorder that affects movement. Motor symptoms like stiffness, bradykinesia (slowed movement), tremors, and postural instability are brought on by this dopamine insufficiency. Non-motor symptoms like cognitive decline and emotional disorders are also linked to PD. Lewy bodies, clumps of the protein alpha-synuclein, are the distinguishing feature of PD. Although hereditary factors and exposure to environmental chemicals have been linked to PD, its specific origin is still unknown [10].

Amyotrophic Lateral Sclerosis

The motor neuron ailment ALS, often known as Lou Gehrig's disease, causes both upper and lower motor neurons to deteriorate progressively. This leads to eventual respiratory failure, muscular weakening, and paralysis. Most occurrences of ALS are random; however, a small percentage are inherited because of mutations in genes like SOD1 and C9orf72. Neurodegeneration in ALS is caused by incorrect protein folding, compromised axonal transport, and activated glial cells [11].

Alzheimer's Disease

AD accounts for 60-70% of all occurrences of dementia, making it the most prominent cause. The buildup of tau protein tangles and beta-amyloid plaques in the brain distinguishes it. Memory loss, cognitive deterioration, and behavioral changes are caused by these aberrant protein aggregates, which interfere with neural communication [12].

The gut-brain axis: mechanism of communication

The CNS and GI system interact with one another in an intricate network known as the gut-brain axis. It produces a dynamic connection between the microorganisms in the GI tract, the brain, and those microorganisms. This axis is composed of numerous complex pathways, including microbial, neurological, hormonal, and immunological ones. Together, these routes support constant information flow. In addition to digestion and nutritional absorption, this interplay has effects on a variety of physiological processes, such as mood regulation, cognition, immune response regulation, and even behavior [13,14].

Neural Pathways

The gut-brain axis is built on the vagus nerve, a crucial component of the autonomic nervous system that establishes a direct neurological connection between the gut and the brain [15]. The vagus nerve, which serves as a bidirectional channel, transmits signals from the gut to the brain in both directions. The neurotransmitters serotonin, gamma-aminobutyric acid (GABA), and dopamine are created in the gut and are crucial for controlling mood and behavior. The sophisticated neural network in the GI tract known as the enteric nervous system (ENS), the "second brain," works closely but independently with the CNS.

Hormonal Pathways

Another crucial pathway for communication between the gut and brain is hormonal signaling. Enteroendocrine cells, found throughout the digestive system, release hormones in response to various stimuli, such as the presence of nutrients or metabolites made by the gut microbiota. Ghrelin, glucagon-like peptide-1, and cholecystokinin are a few of the hormones that control hunger, satiety, and energy levels. In addition to affecting metabolic functions, gut hormones can also affect how we think and feel [8].

Microbial Pathways

The part the gut microbiota plays in the gut-brain axis is arguably the most intriguing. These microscopic creatures weigh between 1 and 2 kg and can produce a fantastic variety of bioactive chemicals. Neurotransmitters, SCFAs, and other metabolites produced by gut bacteria can directly impact brain activity and behavior. For instance, certain gut bacteria can create neurotransmitters like dopamine and serotonin, which can influence mood and emotional reactions. Additionally, microbial metabolites can alter the BBB's integrity, affecting how molecules travel from the gut to the brain [16].

Gut microbiota dysbiosis in neurodegenerative diseases

It has received a lot of attention that gut microbiota dysbiosis, which is characterized by an imbalance in the makeup and function of the gut microbial population, may have a role in the pathogenesis and development of a number of neurodegenerative illnesses.

Dysbiosis and Inflammation

Chronic inflammation in the brain is frequently linked to neurodegenerative illnesses like AD, PD, and ALS. Through a number of methods, dysbiosis in the gut microbiota can foster an inflammatory milieu. Inflammation throughout the body can result from metabolites that dysbiotic microorganisms can generate. Additionally, a dysbiosis-related disruption in gut barrier function can cause microbial compounds to leak into the bloodstream, setting off an inflammatory cascade [17,18].

Metabolite Production

Numerous metabolites, such as SCFAs and neuroactive substances, are abundant in the gut microbiota. It has been demonstrated that the SCFAs produced by gut bacteria during the fermentation of dietary fibers have neuroprotective qualities. They can control the generation of neurotransmitters, the regulation of immunological responses, and BBB integrity. Dysbiosis can change the equilibrium of SCFAs, which could affect their advantageous effects on brain health. Some gut bacteria can also create neuroactive substances like GABA and serotonin, which are vital for controlling mood and maintaining brain health. Dysbiosis-related dysregulated synthesis of these substances may be a factor in the development of neurodegenerative diseases [19,20].

Protein Misfolding and Aggregation

According to a growing body of studies, unfolded proteins linked to neurodegenerative illnesses may move from the gut to the brain via prion-like pathways. For instance, in PD, aggregates of misfolded alpha-synuclein have been discovered in the ENS before reaching the brain. Dysbiosis aids in this process by encouraging the accumulation of misfolded proteins and the sequent transport to the brain. The complex connections between gut health, protein aggregation, and neurodegeneration are highlighted by this phenomenon [21].

Microbiota-brain signaling and neuroinflammation

One of the important mechanisms by which the gut microbiota influences neurodegenerative diseases is by orchestrating microbiota-brain signaling, which can contribute to neuroinflammation, a hallmark feature of these disorders. The complex communication network between the gut and the brain, known as the gut-brain axis, plays a pivotal role in the modulation of various physiological and pathological processes [22].

Neuroinflammation and Neurodegeneration

Microglia are activated during neuroinflammation, a complicated immune response affecting the CNS. Inflammatory mediators such as cytokines and chemokines are also released. The pathogenesis of neurodegenerative illnesses, including AD, PD, and ALS, is thought to be influenced by chronic and severe neuroinflammation, although inflammation is a natural protective response. Microglia, the brain's native immune cells, can over-activate in conditions that cause neurodegenerative illnesses and release neurotoxic compounds that cause cell death and neuronal damage [23,24].

Microbiota-Induced Neuroinflammation

Signals coming from the gut can affect brain function and vice versa through the gut-brain axis, which acts as a bidirectional communication channel. An imbalance in the gut microbial community known as dysbiosis can start signaling pathways that support neuroinflammation. Lipopolysaccharides and other compounds released by dysbiotic microorganisms can breach the gut barrier and activate immune cells in the gut-associated lymphoid tissue. Pro-inflammatory cytokines and other signaling molecules are released as a result of this activation, and they can go to the brain via a variety of paths, including neurological and circulatory pathways [25,15].

Immune Cell Migration and Activation

Immune cells move and get activated due to dysbiosis, causing neuroinflammation in the brain. Immune cells and chemicals can go through the damaged intestinal barrier and into the bloodstream and then travel to the brain. In response to inflammatory signals, peripheral immune cells such as monocytes and T cells can invade the brain. These immune cells then contribute to the neuroinflammatory response in the brain by releasing cytokines, chemokines, and other pro-inflammatory substances that keep the cycle of inflammation and neuronal injury going [26].

Gut Microbial Metabolites

SCFAs, a type of metabolite generated by the gut microbiota, are crucial for the communication between the microbiota and the brain. SCFAs can affect the synthesis and release of neurotransmitters, BBB integrity, and immune cell activity. The production and availability of these metabolites can be altered by dysbiosis, interfering with their beneficial effects on the control of neuroinflammation and general brain health [27].

Implications for disease progression and onset

Beyond simple connections, the complex relationship between the gut microbiota and neurodegenerative disorders has important ramifications for the beginning and development of these crippling ailments. The complicated interplay that can either increase disease pathogenesis or provide opportunities for therapeutic approaches is made possible by the bidirectional communication along the gut-brain axis.

Disease Progression and Aggregation

The gut microbiota's effects can strongly impact the course of the disease on protein misfolding and aggregation, two symptoms of neurodegenerative diseases. It is possible that misfolded proteins, such as beta-amyloid and tau in AD and alpha-synuclein in PD, start in the stomach before spreading to the brain. These proteins may aggregate in the ENS due to changes in the gut environment brought on by dysbiosis, making it easier for them to travel through neural pathways to the brain. This phenomenon indicates the possible function of the gut microbiota in triggering or amplifying neurodegenerative processes and potentially hastening the progression of the disease [28].

Early Biomarkers and Diagnostics

The impact of the gut microbiota on neurodegenerative illnesses may open up new possibilities for early identification and treatment. Recent research indicates that alterations in the mix of gut bacteria and metabolite profiles may be used as indicators for neurodegenerative disorders. Researchers could isolate different microbial fingerprints connected to illness states by examining the gut microbiota. These microbial indicators allow for the early detection of those at risk of neurodegenerative illnesses, allowing for prompt therapeutic interventions and individualized treatment plans [29].

Therapeutic Interventions

The gut microbiome significantly impacts neurodegenerative illnesses, which opens up novel therapy options. Slowing disease onset can be slowed by altering the gut microbiota through dietary changes, probiotics, prebiotics, and postbiotics. It might lessen neuroinflammation, enhance neurotransmitter balance, and impact protein misfolding by encouraging a gut environment rich in advantageous microorganisms. Additionally, methods to increase the production of neuroprotective metabolites, including SCFAs, may provide neuroprotection and postpone the start of the disease [30,31].

Therapeutic approaches and future directions

A paradigm change in therapy strategies and new avenues for future study have been made possible by the complex interactions between gut microbiota and neurodegenerative disorders. Utilizing the gut-brain axis's two-way connection offers new ways to slow disease progression, enhance patient outcomes, and even change how neurodegenerative diseases are managed.

Microbiota Modulation for Neuroprotection

Increasing data points to the potential for neuroprotection offered by targeted manipulation of the gut microbiota. The development of advantageous microbial species that generate neuroprotective compounds may be encouraged by methods for reestablishing a healthy gut microbial balance, such as dietary interventions high in fiber and prebiotics. These substances, which include SCFAs and other bioactive chemicals, may help to preserve cognitive function, keep the brain healthy, and prevent the beginning of neurodegenerative illnesses [32,33].

Development of Probiotics and Postbiotics

Live microorganisms known as probiotics are being studied as treatment options for neurodegenerative illnesses because of their potential health advantages. Specific probiotic strains may influence the gut-brain axis, lessen inflammation, and improve the balance of neurotransmitters, according to research. Postbiotics, bioactive substances created by probiotics during fermentation, also show promise since they can deliver comparable advantages without requiring live microorganisms [34].

Biomarker Discovery and Personalized Medicine

The identification of biomarkers that can forecast risk factors for illness, development, and therapy response is made possible by developments in microbiome research. Diagnostic tools and individualized treatment plans may be created by discovering microbial signatures linked to particular neurodegenerative disorders. These methods may improve early diagnosis and allow customized therapies based on each person's unique gut microbiota patterns [35].

## Conclusions

The complex interaction between gut microbiota and neurodegenerative disorders has revealed a fascinating new area of medical research. The complex interactions between the gut and the brain have shed light on the possibility of using the microbiota as a potent moderator of illness development and progression. The importance of gut microbiota in determining brain health is underscored by the research surrounding its effects on inflammation, protein misfolding, neurotransmitter abnormalities, and more. The prospects for this field in the future are both exciting and difficult. The therapeutic potential of microbiota-based approaches may transform the management of neurodegenerative diseases. Advanced treatments that focus on many elements of disease etiology are made possible by precise microbiota interventions, creative delivery techniques, personalized courses, and multi-omics integration. The discovery of biomarkers and the creation of ethical and legal frameworks become crucial as researchers attempt to understand the complex pathways connecting the gut and the brain. This trip has its challenges, though. For microbiota-based therapeutics to be shown to be safe and effective, rigorous clinical trials are essential. Careful maneuvering is needed to balance the ethical issues of patient autonomy, fair access, and potential hazards. Transforming scientific discoveries into practical applications that help patients will require interdisciplinary cooperation. Additionally, considering the complexity of neurodegenerative illnesses necessitates multiple strategies that consider patient groups' variation. The gut microbiota stands out as a remarkable participant in the larger picture of neurological health, with the ability to influence disease trajectories and rethink therapy approaches. Researchers and doctors are in a prime position to find ground-breaking treatments that provide hope for those suffering from neurodegenerative diseases by comprehending and utilizing the strength of this deep link. It is becoming increasingly obvious as science develops that the gut-brain axis holds the key to opening up fresh therapeutic possibilities and revolutionizing the field of study and treatment of neurodegenerative diseases. However, the study has certain limitations, such as the fact that most of the research was conducted on animal models, which may not directly translate to humans. Thus, more research and sampling must be done to confirm the findings in human bodies.
